# The Cupola: an additional layer of protection for providers working in the oropharyngeal region

**DOI:** 10.1186/s13104-021-05524-9

**Published:** 2021-03-25

**Authors:** Alessandro Villa, Marlene Grenon

**Affiliations:** 1 Department of Orofacial Sciences, University of California San Francisco, 513 Parnassus Ave, Suite 512A, San Francisco, CA 94143 USA; 2 Innovation Ventures, University of California San Francisco. San Francisco, San Francisco, CA USA; 3 Department of Surgery, University of California San Francisco, San Francisco, CA USA

**Keywords:** COVID-19, Aerosol, Shield

## Abstract

**Objectives:**

To reduce the spread of the infection, especially during aerosol generating procedures, we invented “The Cupola”, a shield that creates a mechanical barrier around the patient’s head and body. With this pilot study we aimed to assess the effectiveness of an additional layer of protection (The Cupola) developed for providers working in the oropharyngeal region.

**Results:**

The mean number of 0.3 μm particles with no Cupola was 3777 (SD: ± 556), with The Cupola was 2068 (SD: ± 1468) and with the Cupola and Drape was 2031 (SD: ± 1108) (p < 0.015). The mean number of 0.5 μm airborne particles with no Cupola was 65 (SD: ± 7), with The Cupola was 29 (SD: ± 28) and with the Cupola and Drape was 28 (SD: ± 23) (p < 0.05). Results showed a significant reduction of aerosols generated during simulated dental procedures when the Cupola was used. The Cupola offers an extra layer of protection in addition to the recommended personal protective equipment.

## Introduction

Even though most countries have begun to allow the delivery of dental and medical services, providing safe oral healthcare routinely remains challenging due to the high transmissibility of the Severe acute respiratory syndrome coronavirus 2 (SARS-CoV-2 virus; coronavirus) and how easily it may be dispersed during aerosol-generating procedures [1]. SARS-CoV-2 is present in salivary and nasopharyngeal secretions of infected patients, and spreads through respiratory droplets (droplet nuclei less < 5 μm in size) as well as small viral particles that can linger in the air [2, 3].

Global Coronavirus disease (or COVID-19) cases have surpassed 115 millions [4]. The COVID-19 pandemic is placing health care professionals performing aerosol-generating procedures in the oropharyngeal region at risk for becoming infected and subsequently, become vectors of infection [5]. As such, patients, and especially vulnerable individuals for COVID-19 may hold off from obtaining dental care in view of the perceived risk of such visits and procedures [6]. The Health Policy Institute of the American Dental Association showed a possible 38% decline in dental care spending in the U.S. in 2020 and 20% in 2021. In October, 2020 more than a half of dental practices were open with a lower patient volume (55.2%) than usual [7].

Poor access to dental care can lead to untreated dental decay or other oral health infections, leaving people with no viable options other than visiting hospital emergency rooms, where treatment is costly and disrupts more urgent care needs, particularly in a time of crisis such as the current COVID-19 pandemic [8]. Additionally, missed routine dental visits represent failed opportunities to provide preventive oral health care and to identify oral manifestations of systemic diseases [9].

**Fig. 1:**
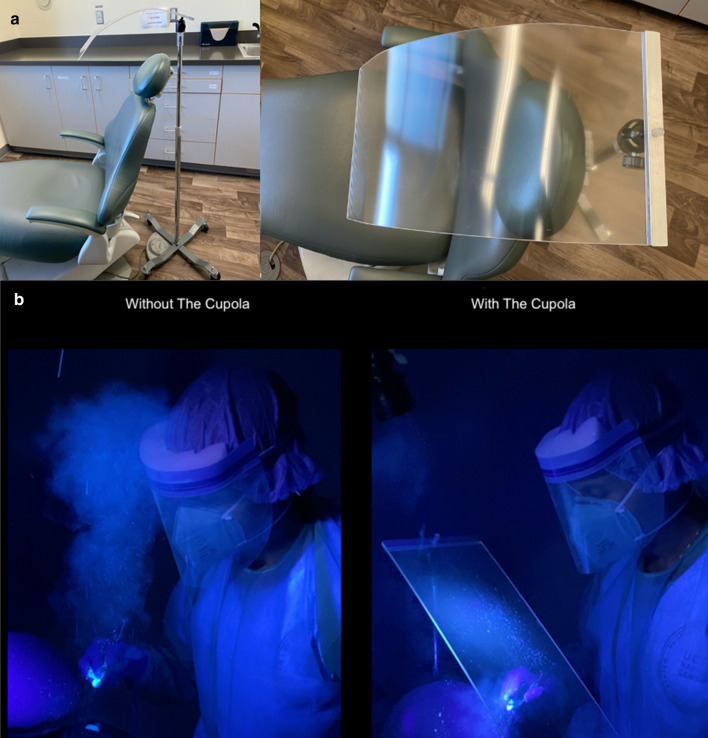
**a** The Cupola—protective shield. **b** Simulated dental procedure using a high-speed handpiece without and with The Cupola

To reduce the spread of the infection, especially during aerosol generating procedures, we invented “The Cupola”, a shield that creates a mechanical barrier around the patient’s head and body, and allows health providers to perform their work unhindered (Fig. 1a)[10]. “The Cupola” includes a face shield on a cart (or anchored to the dental chair) and a disposable waterproof surgical utility drape with tape around the shield to cover the patient head and body (as an additional option). The shield can be reused after disinfection with an Environmental Protection Agency (EPA)-registered, hospital-grade disinfectant.

We hypothesized that the use of The Cupola during aerosol generating procedures in the oropharyngeal region decreases the spread of droplets and aerosols in dental offices, which could lead to safer dental practices. This has potential implications to decrease the spread of disease among dentists, maxillofacial surgeons, otolaryngologists and their patients. The aim of this pilot study is to assess the efficacy of The Cupola in decreasing the spread of droplets and aerosols during simulated aerosol generating dental procedures.

## Main text

### Macroscopic contamination

All experiments were carried out by one of the investigators (A.V.) in a dental office. We used Glo-germ™ (Glo Germ Company, Moab, UT, USA), a fluorescent resin powder with particle sizes between 1 and 5 µm (SARS-COV-2 is 0.07–1.2 µm), with ultraviolet light detection in a darkened dental operatory [11]. Glo-germ™ was placed into the water lines of the dental chair. Between experiments, the dental chair and surfaces were cleaned with alcohol swipes. All experiments were video recorded using an iPhone 11 (Apple Inc, Cupertino, CA, USA). A simulated dental procedure using a high-speed handpiece was performed without and with The Cupola and repeated for five times. We showed that aerosols generated by the high-speed handpiece were limited when the Cupola was used (Fig. 1b). Specifically, the experiments without The Cupola revealed macroscopic contamination around dental chair and on the face shield of the provider, while during the experiments with The Cupola there was contamination of the inner surface of The Cupola and the provider’s gloves.

### Microscopic contamination

We used a high-speed handpiece for 1 min and repeated the experiment three times without The Cupola, with the Cupola and with The Cupola and drape. Between experiments, the dental chair and surfaces were cleaned with alcohol swipes. The Lighthouse 3016IAQ airborne particle counter (Fremont, CA) was positioned on a tray pre-set at head height (50 cm above the dental chair headrest) immediately in front of the provider’s head. This height of the airborne particle counter was maintained throughout the duration of the experiment. The airborne particle counter, typically used for indoor air quality testing in semiconductor cleanrooms, research laboratories and operating theatres, utilizes a laser diode and photo detector to count particles by collecting scattered light from particles as they pass through a sample inlet. The device counts airborne particles of 0.3, 0.5, 1.0, 2.5 and 5.0 microns. Airborne particle counter flow rate was set to 2.83 l min^−1^ with detection of the ambient air occurring in 1 s sweeps. All values were normalized against the background particle count present in the room at the start of each recording.

### Analyses and results

We calculated the mean number of airborne particles for all five experiments without The Cupola, with the Cupola and with The Cupola and drape. Differences between the three groups were calculated using the Nonparametric Wilcoxon Test at 1-min intervals [12]. All statistical analyses were carried out using JMP®, Version 15; SAS Institute Inc., Cary, NC. Statistical significance was defined as p < 0.05.

**Fig. 2:**
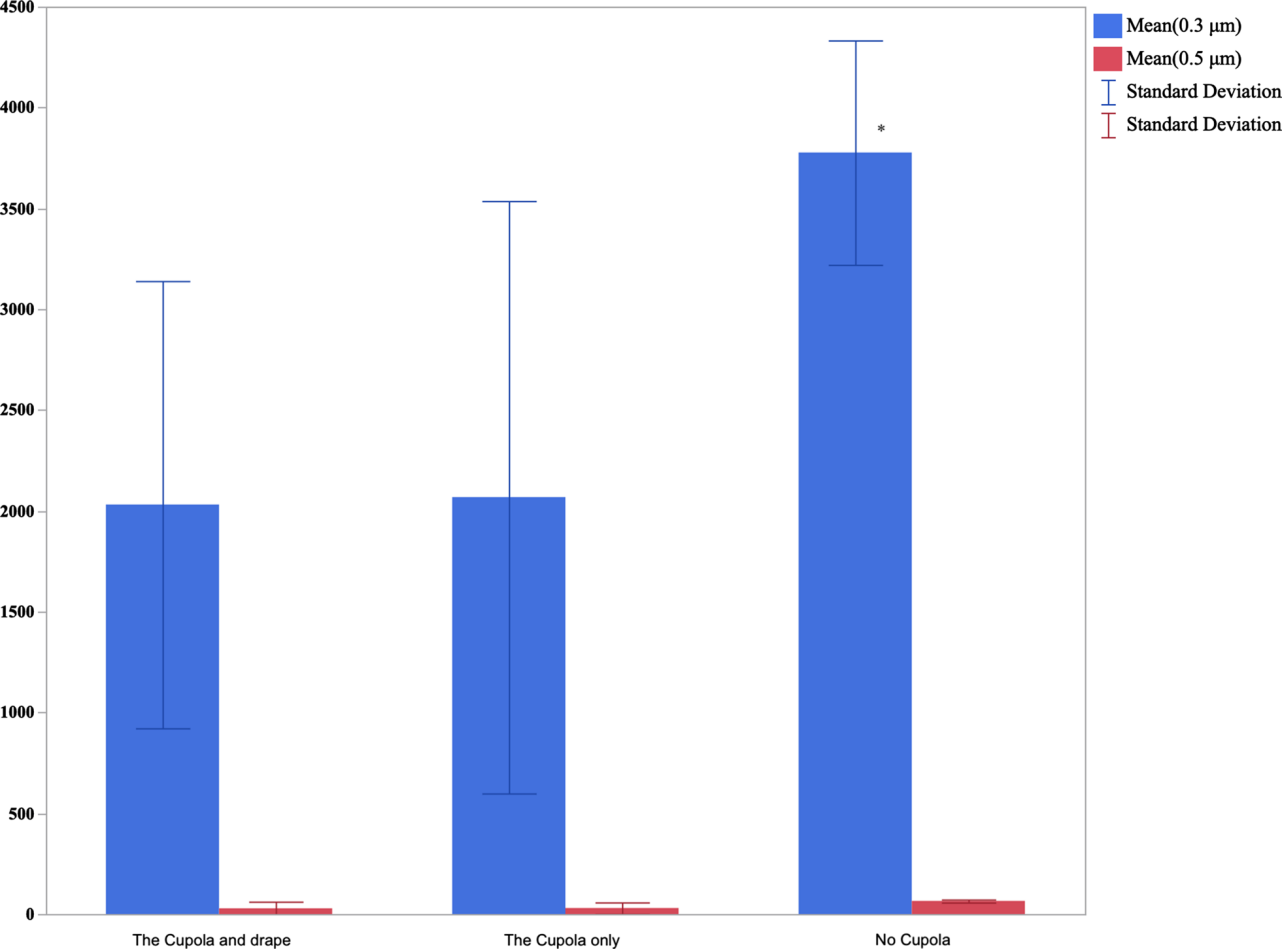
Mean number of particles with The Cupola, with The Cupola and the drape, and without The Cupola

The mean number of 0.3 μm particles with no Cupola was 3777 (SD: ± 556), with The Cupola was 2068 (SD: ± 1468) and with the Cupola and Drape was 2031 (SD: ± 1108) (p < 0.015). The mean number of 0.5 μm airborne particles with no Cupola was 65 (SD: ± 7), with The Cupola was 29 (SD: ± 28) and with the Cupola and Drape was 28 (SD: ± 23) (p < 0.05; Fig. 2).

### Conclusion

We have shown that The Cupola is effective at decreasing aerosols and droplets generated during simulated dental procedures. With the current pandemic, there is a need for alternate methods to reduce exposure of health care providers to pathogens present in a patient’s oral cavity and airways (provider’s protection). Even after the COVID-19 pandemic, professionals will continue to be exposed to existing and possible new pathogens. The Cupola provides an additional layer of protection against such pathogens. Several porotypes have been developed over the past few months to include an articulating arm with a clamp for attaching to a headrest or backrest of a dental chair or operating bed. We believe The Cupola will support dental providers and other physicians working in the head and neck area in returning to perform operations with a volume of visits closer to pre-COVID-19 pandemic levels`, while keeping staff, patients and themselves safe and allowing opportunities for oral disease management.

## Limitations

One of the limitations of the study was that the aerosol generated procedures were simulated and not on patients. However, the spread of the macroscopic particles may have been even higher because the mouth itself may create a mechanical barrier during dental procedures with high speed instruments. In addition, during most aerosol generating dental procedures with a high-speed hand piece, dentists typically use a rubber dam isolation and/or high-power vacuum suction which may also markedly reduce the spread of virus-contaminated particles.

## Data Availability

Not applicable.
